# Replacing ADT With dTpa in Queensland Emergency Departments: Another Opportunity for Pertussis Control?

**DOI:** 10.1111/1742-6723.70228

**Published:** 2026-02-23

**Authors:** Michael D. Nissen, Faye Jordan, Polash Adhikari, Sebastian Vernal, Luis Furuya‐Kanamori

**Affiliations:** ^1^ The Prince Charles Hospital and Royal Brisbane and Women's Hospital Metro North Health Brisbane Queensland Australia; ^2^ UQ Centre for Clinical Research, Faculty of Health, Medicine and Behavioural Sciences The University of Queensland Brisbane Queensland Australia; ^3^ The Prince Charles Hospital Metro North Health Brisbane Queensland Australia

The recent pertussis resurgence in Queensland underscores a critical missed opportunity in adult immunisation. With 15,030 cases reported in 2024, over 16 times the number of the previous year, the epidemic included over 6800 infections in school‐aged children and nearly 200 hospitalisations, tragically including at least one two‐month‐old infant fatality [1]. This resurgence, likely fuelled by declining maternal pertussis coverage, waning population immunity and insufficient adult booster uptake, demands urgent action. Pertussis vaccination among pregnant women fell from 77.2% in 2020 to 70.7% in 2023, with some regions below 65% coverage [2]. Decreased immunity following acellular‐pertussis vaccination compared to whole‐cell pertussis vaccination in infancy has also been linked to increased pertussis incidence in school‐aged children [3]. One underutilised yet immediately actionable strategy is the substitution of dTpa (reduced antigen diphtheria‐tetanus‐acellular pertussis) vaccine for ADT (adult diphtheria‐tetanus) in emergency department (ED) wound management protocols.

While ADT remains a cornerstone of tetanus prophylaxis in wound management, it offers no protection against pertussis. Conversely, dTpa provides equivalent protection against tetanus and diphtheria, while also inducing immunity against pertussis lasting up to 4 years [4]. In practice, substituting dTpa for ADT simply broadens the benefit without requiring any additional procedures. Notably, the Australian Immunisation Handbook authorises the use of a pertussis‐containing vaccine for adult tetanus prophylaxis in wound care [5]. Despite this, ADT remains the default in many Queensland EDs. In 2024, a total of 72,980 doses of ADT were distributed to Queensland Health Services Districts [6]. This status quo reflects an inertia in practice rather than evidence‐based policy. When pertussis is spreading widely in communities and disproportionately affecting young children, failing to capitalise on this immunisation opportunity in EDs is an avoidable oversight.

Emergency departments are uniquely positioned to deliver opportunistic adult vaccinations. They are the first point of care for traumatic injuries, minor wounds and tetanus‐prone presentations, scenarios where tetanus‐containing vaccines are routinely administered. In these cases, switching from ADT to dTpa involves no change to clinical workflow, staffing, or logistics, only a substitution of vaccine product. The vaccine is delivered intramuscularly, requires the same consent and observation period, and is commonly stocked in hospital formularies. This makes ED‐based dTpa delivery one of the lowest‐hanging fruits in public health response.

Scientific evidence supports the clinical rationale. Adults play a central role in pertussis transmission. Among infants for whom a source can be identified, approximately 76%–83% acquire pertussis from household contacts, most commonly a parent [7]. Adult pertussis is frequently underdiagnosed and may present as a persistent cough or be mistaken for other respiratory conditions. Nevertheless, infected adults remain contagious for weeks and contribute to community spread, including seeding outbreaks in schools, workplaces and households. Opportunistic immunisation of adults, such as during an ED visit, reduces the risk that an asymptomatic or mildly symptomatic parent or caregiver will unknowingly transmit pertussis to an infant.

Protection against pertussis is not lifelong. Immunity from childhood vaccination—particularly with acellular pertussis vaccines introduced in the late 1990s, wanes over time [3, 5]. Even fully immunised children may have substantially reduced protection within 4 years [8]. Without a booster, many individuals become susceptible again by adolescence or adulthood, contributing to cyclic pertussis epidemics every few years [5]. Many Queensland adults likely have not received a pertussis‐containing vaccine since childhood, if ever, making an ED visit a rare and valuable opportunity to restore protection.

The immunogenicity of dTpa in adults is well established. A single dose elicits robust antibody responses against all three components (diphtheria, tetanus, pertussis), with seroprotection persisting for several years [5]. Clinical trials and post‐marketing surveillance have demonstrated the safety profile of dTpa to be comparable to that of ADT. Common adverse events are mild and self‐limited (e.g., local soreness, fatigue), and serious events are rare. Furthermore, dTpa is endorsed by the Australian Technical Advisory Group on Immunisation (ATAGI) for adult booster use and is included in the Australian Immunisation Handbook for use in wound management [5].

Internationally, emergency settings have increasingly been recognised as strategic vaccination touchpoints. In the United States, the Advisory Committee on Immunization Practices (ACIP) explicitly recommends the use of Tdap (the US equivalent of dTpa) in adults requiring a tetanus booster for wound management if they have never received a pertussis‐containing vaccine [4]. United Kingdom guidelines similarly encourage opportunistic adult vaccination in clinical settings [9]. Several Australian ED audits and commentaries, report missed opportunities for adult immunisation, especially around tetanus prophylaxis in wound and laceration care and inadequate screening for other adult vaccines [10, 11].

The barriers to implementing this change are minimal and largely perceptual. Concerns about cost or supply are not supported by current evidence. The approximate difference in vaccine cost is currently an extra A$6 per dose [12]. The direct primary‐care cost of adult pertussis is about A$473–A$909 per case [13], so preventing cases has clear public health value. There may be a need for initial staff education to address clinical inertia, but no structural changes are required. Both vaccines are stocked in Queensland public hospitals, and both have clear clinical guidelines [5]. Moreover, pharmacists and nursing staff already trained to administer ADT require no retraining for dTpa. ED clinicians have adopted dTpa in place of ADT when institutional protocols standardised wound care boosters [14].

It is suggested that vaccines with the pertussis component are unpopular or resisted by patients. However, current data does not support this. Rather, the shift in vaccine sentiment appears to be broader and pandemic related. Declines in immunisation coverage across Australia have been documented across multiple childhood vaccines, including measles, polio and pertussis [15]. This is not necessarily due to pertussis‐specific concerns, but part of a more generalised vaccine hesitancy phenomenon emerging in the post‐COVID era. Surveys and behavioural research indicate a rise in mistrust of public health recommendations, increased misinformation through social media and declining on‐time childhood vaccination even among previously compliant populations [16]. A recent national study conducted across eight US cities confirmed that underserved emergency department populations remain open to opportunistic vaccination offers when screening is embedded in clinical practice [17]. Thus, vaccine hesitancy today is not targeted at pertussis‐containing vaccines alone but reflects a broader erosion in public confidence across all immunisation efforts.

What is urgently needed is an institutional and policy‐level commitment to embed dTpa as the default ‘tetanus shot’ in standard practice. Queensland Health could lead with a directive outlining clear steps to replace ADT with dTpa for adults requiring tetanus prophylaxis, particularly during periods of pertussis resurgence. Local Health and Hospital Services could include dTpa in standing orders, wound management pathways and ED electronic medical record prompts. Hospital administration and pharmacy departments should be engaged to drive this change through formulary management and local policy updates. Primary Health Networks could also be empowered to promote this substitution within general practices and Medicare‐funded urgent care centres.

It is especially important to consider vulnerable groups, such as Aboriginal and Torres Strait Islander peoples, who are more likely to present late, have a higher disease burden and face barriers to follow‐up care. Likewise, rural and remote residents and individuals from lower socio‐economic backgrounds are more likely to seek care through EDs. ED‐based vaccination thus offers a practical and equitable means to reach populations who may not access regular immunisation services. Opportunistic pertussis vaccination in this setting provides a rare opportunity to close immunity gaps and protect both individuals and their broader communities.

This opinion is not a call for routine adult dTpa vaccination via EDs for all comers; rather, it targets a narrow, well‐defined cohort already receiving a tetanus vaccine. This precision allows for a high‐yield intervention with minimal marginal effort. Each ADT dose administered instead of dTpa in this context represents a missed opportunity for pertussis prevention, a potential infant exposure averted, and a contribution to community immunity.

In conclusion, the replacement of ADT with dTpa in emergency departments represents a clinically sound, logistically feasible and ethically responsible strategy. This strategy is crucial because it leverages every healthcare encounter to prevent disease—particularly in the context of resurgent vaccine‐preventable illnesses such as pertussis. By incorporating pertussis boosters into routine ED practice, clinicians can make a meaningful contribution to public health without altering existing workflows. It capitalises on an existing clinical workflow, responds to a pressing public health threat and positions emergency clinicians as partners in vaccine‐preventable disease control. As Queensland recovers from an unprecedented pertussis resurgence, implementing this simple intervention could help delay or reduce the severity of future epidemics by enhancing population‐level immunity. Failure to act would disregard expert immunisation advice, clinical and epidemiological evidence and would represent a missed opportunity to protect the very communities emergency medicine serves.

## Author Contributions

All authors contributed equally to the conceptualisation, drafting and revision of the manuscript.

## Funding

The authors have nothing to report.

## Conflicts of Interest

The authors declare no conflicts of interest.

## Data Availability

Data sharing not applicable to this article as no datasets were generated or analyzed during the current study.
